# Supplementary material for: Single-cell and extracellular nano-vesicles biosensing through phase spectral analysis of optical fiber tweezers back-scattering signals
Source: Commun Eng. 2024 Jul 10;3:97. doi: 10.1038/s44172-024-00240-1 (PMC11236981; doi:10.1038/s44172-024-00240-1)
Supplement: Supplementary file 2 — Supplementary Information [file 44172_2024_240_MOESM2_ESM.pdf]

Single-cell and Extracellular Nano-Vesicles Biosensing through Phase Spectral Analysis of Optical Fiber Tweezers Back-scattering signals

Beatriz J. Barros[1] , João P. S. Cunha[1,2] (jcunha@ieee.org)

1 - INESC TEC - Institute for Systems and Computer Engineering, Technology and Science, Porto, Portugal

2 – Faculty of Engineering, University of Porto, Porto, Portugal

SUPPLEMENTARY INFORMATION

Supplementary Note 1: Validation Experiments

Considering the reduced amount of literature on phase calculation and unwrapping, the corresponding processes were tested through a series of validation experiments involving synthetic signals. To verify the phase unwrapping algorithm applied, a sinusoidal phase signal was created, exceeding the [$-\pi$, π] limit in order to generate discontinuities during the phase spectrum calculation. This is observed in Supplementary Figure 1 (A-B). The unwrapping procedure was conducted manually for each discontinuity, by transversing in the x direction through the wrapped phase vector to detect the presence of discontinuities between adjacent samples, that are compensated with a 2π addition or 2π subtraction when a difference larger than $+\pi$ or smaller than $-\pi$, respectively. This processing is illustrated in Supplementary Figure 1 (C). The final result was then compared with the phase spectrum retrieved from unwrap built-in MATLAB function (Supplementary Figure 1 (D). The original phase was perfectly retrieved which demonstrated the suitability of the algorithm to remove the discontinuities and obtain a continuous phase signal.

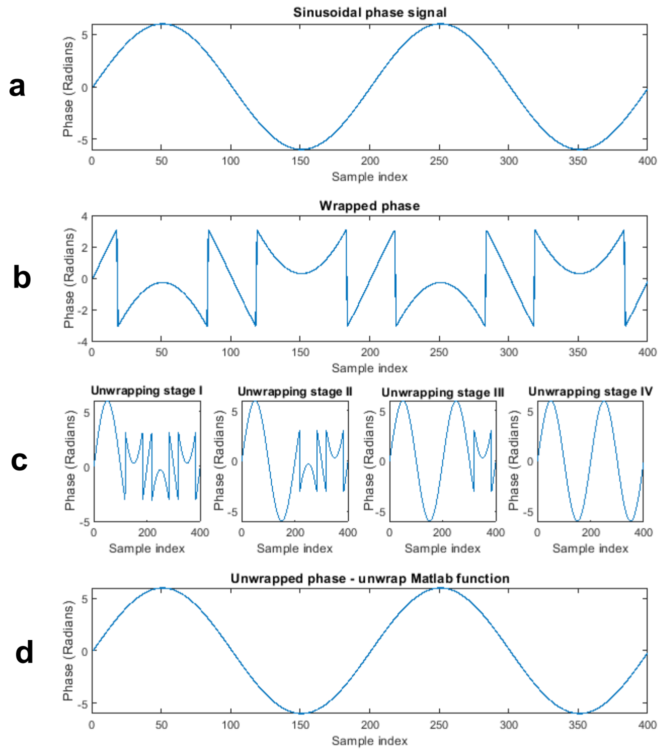

Supplementary Figure 1 Illustration of the phase unwrapping validation experiment conducted. a) A sinusoidal phase was created exceeding the [-π, π] limit in order to generate discontinuities during the phase spectrum calculation. b) The wrapped spectrum was obtained as a consequence of phase calculation. c) Unwrapping procedure was calculated manually by compensating the discontinuities found with 2π addition or 2π subtraction until all discontinuities were resolved. d) The final result was compared with the unwrapping procedure applied via the *unwrap* built-in MATLAB function, that retrieved the original phase, showing suitability to be applied in the current problem of phase exploration.

To verify the influence of time shifts in the phase, an approach presented in [1] was followed, based on the analysis of a signal with left-right symmetry. When a signal presents this symmetric property with respect to the origin, the phase is linear and equal to zero. By shifting the signal in time, a change in the slope of the linear phase must be observed. To test this, two variations of the first signal were created, as illustrated in Supplementary Figure 2 (A), were a 0.5 second time delay and advance were introduced. This results in the original signal shifted to the left and to the right, respectively [1]. The spectral representations obtained after applying FFT, small magnitude values removal, phase calculation and unwrapping are presented on Supplementary Figure 2 (B). After phase calculation, the πdiscontinuities present were compensated through the phase unwrapping algorithm. As it was expected, a linear phase response is obtained from the symmetrical time domain waveform. When the peak is centered on sample N/2, it presents zero phase. By shifting in the time domain, a changing in the slope of the linear phase is observed [1]. Therefore, it confirms that the algorithm applied provides a correct phase representation and can then be used to extract phase spectrum from the back-scattered signals.

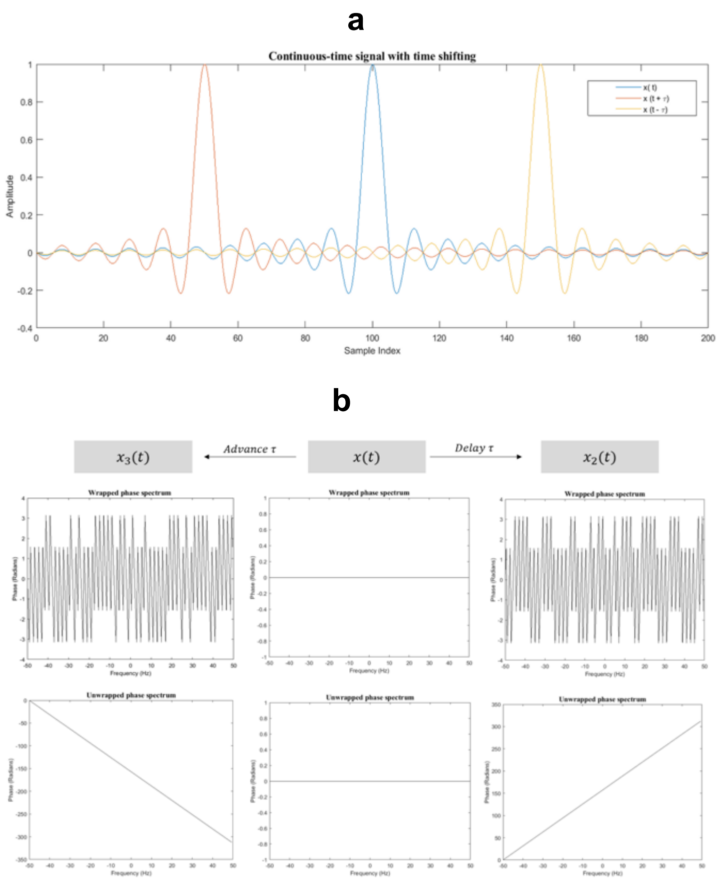

Supplementary Figure 2 Illustration of the phase changes observed with time domain shifts. a) A time domain waveform symmetrical around a vertical axis was selected for this experiment since signals with this type of symmetry present a linear phase spectrum. After phase calculation and unwrapping, a linear phase is obtained as expected for the symmetrical signals used, which confirms that the algorithm applied provides a correct phase representation. b) When the time domain waveform is shifted to the right, the phase remains a straight line, but experiences a decrease in slope. When the time domain is shifted to the left, there is an increase in the slope. Such outcome is equally described for this experiment. This result confirms that the algorithm applied provides a correct phase representation and can then be used to extract phase spectrum from the back-scattered signals. Besides, it illustrates how time-domain shifts influence phase spectrum, thus supporting the hypothesis that phase spectrum can retain patterns related with the bounce-back reflections contained in the back-scattering signal received and analyzed in this experiment.

A new method is presented to obtain phase spectrum – Absolute Hilbert Phase Slope - where phase is calculated at each sampling instant relative to the phase position of the previous point in time, along the horizontal direction (x-direction). To evaluate the suitability of this approach to study phase patterns, a validation experiment was conducted. A synthetic signal was used to calculate the phase of a Hilbert transformed signal (Supplementary Figure 3 (A)). The discontinuities were removed using the previously applied unwrapping algorithm, providing a continuous phase representation, as observed in

Supplementary Figure 3 (B). An interesting observation was registered when calculating the absolute value of unwrapped phase slope. Larger phase shifts were observed in comparison with the wrapped phase wraps observed, that seem to be masked in this calculation (Supplementary Figure 3 (C)). By calculating the phase slope between adjacent points, larger phase shifts were observed, which may be correlated with larger reflections in our experiment. This way, this method seems to provide a resulting spectrum more sensitive and robust to discriminate phase oscillations.

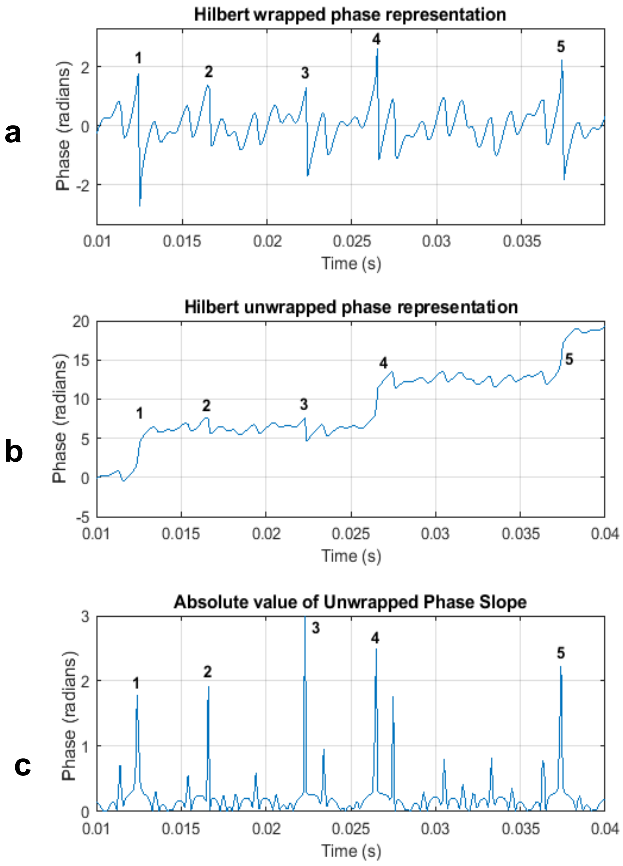

Supplementary Figure 3 Validation experiment for Absolute Hilbert Phase Slope method, where phase is calculated at each sampling instant relative to the phase position of the previous point in time, along the horizontal direction (x-direction). The major phase shifts observed are numerated, for each phase representation: a) Hilbert wrapped phase, b) Hilbert Unwrapped phase and c) Absolute Hilbert Phase Slope. Although phase shifts 4 and 5 seem to present higher amplitude in the first two spectral representations, the absolute unwrapped phase slope revealed new patterns in the phase, where larger phase shifts are identified (e.g., shift 3), that may be hidden in the previous calculation. This result indicates that the algorithm allows a more precise extraction of the phase bounce-back reflection patterns between adjacent points. Therefore, is possible that through this method, light signatures with more detail and specificity are being obtained, creating a new phase representation with considerable improved content in discriminative patterns.

Supplementary Note 2: Mathematical Formulations

Detailed explanation of signal processing procedure with underlying mathematical formulations.

Considering a discrete-time signal with N samples,

$$x(n), n = 0,1,\dots,N-1 \qquad \text{Eq. (1)}$$

The Discrete Fourier Transform is calculated by

$$X[k] = \sum_{n=0}^{N-1} x(n)e^{-j\frac{2\pi nk}{N}},\ k = 0,1,\dots,N-1 \qquad \text{Eq. (2)}$$

where $x(n)$ is the signal value at sample n and $X[k]$ is a sequence of N complex coefficients [2]. This

operation was conducted through the efficient FFT algorithm. For each complex coefficients, the phase parameter was calculated from the inverse tangent function:

$$\emptyset(\omega) = \arctan\left[\frac{Im(X(\omega))}{Re\ (X(\omega))}\right] \qquad \text{Eq. (3)}$$

with Im and Re being the imaginary and real components of the complex Fourier spectrum of the data. This calculation retrieves a phase distribution forced to the principal value range,

$$-\pi < \emptyset(\omega) < \pi \qquad \text{Eq. (4)}$$

Requiring a phase unwrapping algorithm to determine discontinuities on the wrapped phase, resolving them, and achieving a continuous phase spectrum [3]. Mathematically, this phase unwrapping operation is described as [4]:

$$\varphi(x) = \emptyset(x) + 2\pi k(x) \qquad \text{Eq. (5)}$$

where $\varphi(x)$ is the estimated unwrapped phase, $\emptyset(x)$ the wrapped phase obtained from Equation (2), and $k(x)$ is the integer value that specifies the corrective offset required. The procedure is based on transversing, in the x direction, through the wrapped phase vector to detect the presence of discontinuities between adjacent samples, that are compensated with a 2π addition or 2π subtraction when a difference larger than +π or smaller than -π, respectively, is found. This procedure allows removing the discontinuities to obtain a continuous phase signal [4].

The second method used to analyze phase was based on Hilbert Transform (H), related to the Fourier Transform F for the discrete-time signal x as [5], [6]

$$F(H(x))(\omega) = \sigma_H(\omega) + F(x)(\omega) \qquad \text{Eq. (6)}$$

where

$$\sigma_H(\omega) = \begin{cases} i\ for\ \omega < 0 \\ 0\ for\ \omega = 0 \\ -i\ for\ > 0 \end{cases} \qquad \text{Eq. (7)}$$

meaning that negative frequencies are multiplied by i while positive frequencies are multiplied by $-i$, which will produce a 90° rotation in the complex plane, creating a phase-shifted version of the original signal [2]. Hilbert Transform operation allows to calculate instantaneous attributes from the time series $x(n)$, through the computation of the analytical signal [2], [6]:

$$\rho(t) = x_r(t) + jx_i \qquad \text{Eq. (8)}$$

where x_r represents the real part and x_i the imaginary part. This expression in polar form is equivalent to [2], [5]

$$\rho(t) = A(t)e^{j\varphi(t)} \qquad \text{Eq. (9)}$$

where A(t) represents the instantaneous amplitude and $\varphi(t)$ the instantaneous phase, calculated by [2]:

$$\varphi(t) = \arctan\left[\frac{x_i}{x_r}\right] \qquad \text{Eq. (10)}$$

Following the unwrapping procedure presented in Equation (5), the unwrapped instantaneous phase was calculated, and the two types of Hilbert-phase representations explored in this study were obtained: Instantaneous Phase and Instantaneous Phase Slope, calculated respectively by:

$$\varphi_{ABS}(t) = |\varphi(t)| \qquad \text{Eq. (11)}$$

$$\varphi_S(t) = \varphi_n - \varphi_{n-1} \qquad \text{Eq. (12)}$$

Supplementary Note 3: Descriptive statistics formulations

The sub-set of descriptive statistical features is presented in Supplementary Table 1, with measurement specifications and mathematical calculation.

Supplementary Table 1 Back-scattering phase-derived features analyzed.

FEATURE	MEASUREMENT	MATHEMATICAL DESCRIPTION
Standard Deviation	Dispersion	$\sigma = \left(\left(\frac{1}{n-1}\right)\sum_{i=1}^{n}(x_i - \bar{x})^2\right)^{\frac{1}{2}}$
Root mean square	Magnitude of variability	$RMS = \sqrt{\frac{1}{n}\sum_{i=1}^{n} x_i^2}$
Kurtosis	Shape of data distribution	$k = \frac{E(x - \bar{x})^4}{\sigma^4}$
Interquartile Range	Dispersion and dynamic range of the spectrum	$IQR = Q_{75} - Q_{25}$
Skewness	Asymmetry of the samples	$S = \frac{E(x - \bar{x})^3}{\sigma^3}$
Entropy	Structural order of the spectrum	$H = \sum_i p(x_i)$

Supplementary Note 4: Statistical Tests

The class comparison groups evaluated in the statistical analysis stage are schematically presented in Supplementary Figure 4. Each feature was individually evaluated between the selected classes for each problem, considering 4 and 3-class comparisons using Kruskal-Wallis test followed by 2-class comparisons through Mann-Whitney test, with a significance threshold of p-value=0.05 being considered for all tests conducted.

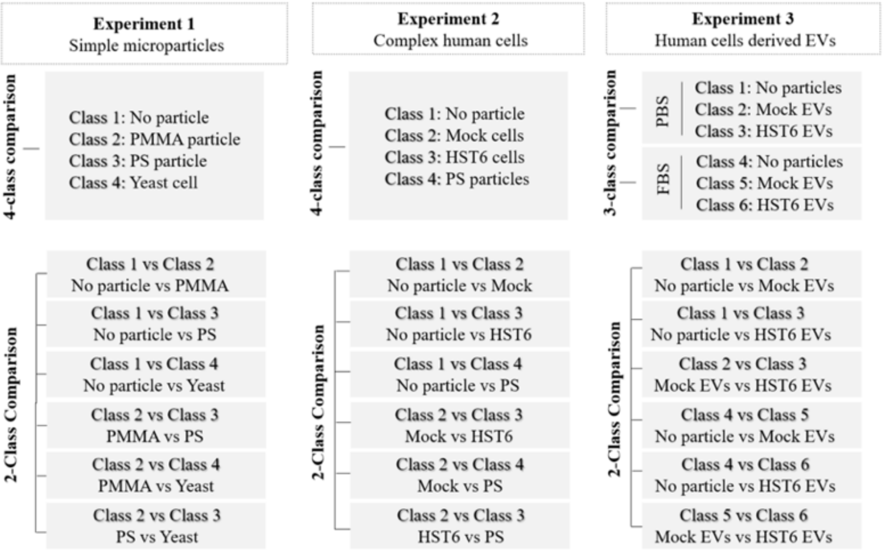

Supplementary Figure 4 Particle class comparisons evaluated in statistical analysis stage.

Supplementary Note 5: 2-class comparisons for Hilbert Phase Slope Coefficients

The results obtained for 2-class statistical comparisons regarding Hilbert Phase Slope coefficients are presented in Supplementary Figure 5, considering experiment 1 with simple microparticles (Supplementary Figure 5 (A-B) and experiment 2 with cancer-derived cells Mock and ST6 (Supplementary Figure 5 (C-D)).

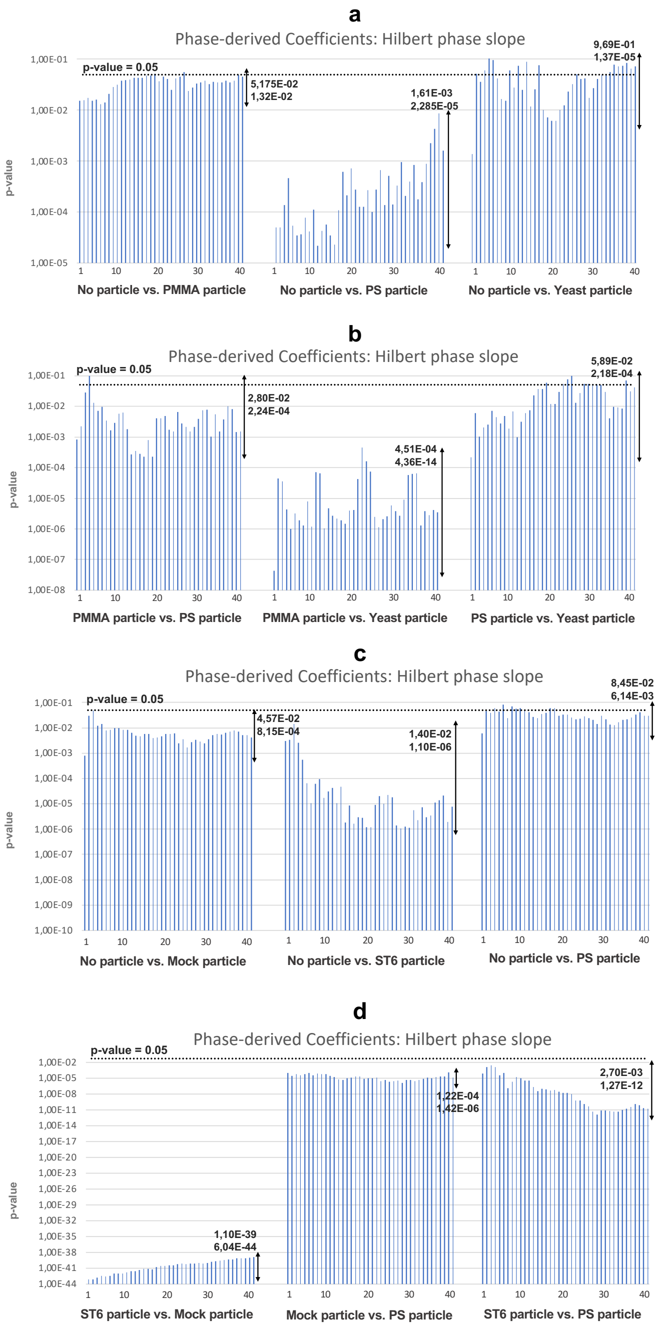

Supplementary Figure 5 Results obtained for 2-class statistical comparison of phase-derived coefficients calculated from Hilbert Phase Slope, regarding Experiment 1 (A – B) and Experiment 2 (C-D). Bars represent the range of p-values obtained in the coefficient group under analysis.

Supplementary Note 6: Surrogate Data Analysis

In order to provide a validation of the statistical results, a surrogate data testing procedure was conducted, to ensure that the corresponding results are a representation of true underlying characteristics of the signals analyzed and exclude the possibility of statistical significance being a consequence of uncorrelated noise [7]. Since the goal was to evaluate the temporal structure contained in the data, a random permutation surrogate was applied. This approach consists of randomly shuffle the original time series, creating a matching dataset where any temporal structure is destroyed [7]. The exact same pre-processing, feature extraction and statistical analysis procedures were conducted, and the results were compared with the original dataset. This comparison is presented in Supplementary Figure 6 for experiment 2 (tumor-derived cells) and Hilbert Phase Slope method, regarding statistical features (Supplementary Figure 6 (A)) and phase-derived coefficients (Supplementary Figure 6 (B)).

a

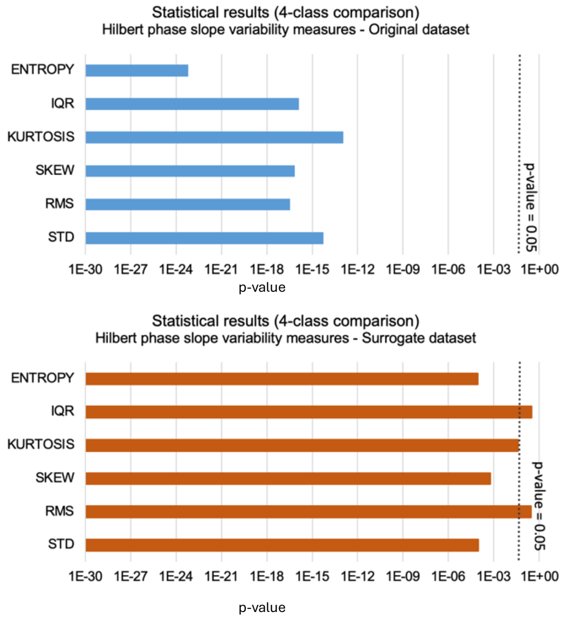

b

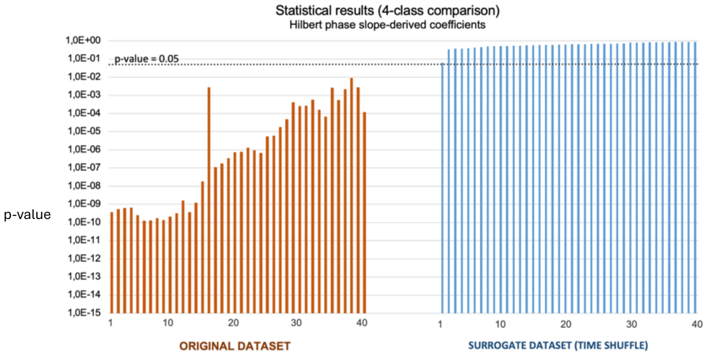

Supplementary Figure 6 Results obtained regarding 4-class statistical analysis of Hilbert phase slope derived features. By destroying the temporal structure of the signals, through the time shuffling algorithm, the set of features no longer presents statistically significant results in the discrimination between the classes of particles. a) Comparison between original and surrogate dataset of statistical features. b) Comparison between original and surrogate dataset of phase-derived coefficients.

The results show that when the temporal structure contained in the phase is destroyed, the features are no longer statistically significant. This strengthens the hypothesis that the light-patterns contained in the phase of the back-scattering signals are responsible for the different light signatures of each particle, that then lead to the statistical significance observed in the correspondent phase-derived features. A

second validation step was conducted only in the Hilbert phase domain obtained for each back-scattering test. After pre-processing, the original phase of each signal portion was replaced for a randomized vector. The feature extraction and statistical analysis procedures were followed exactly in the same manner as the signal processing pipeline previously applied and the results were compared with the original dataset, regarding statistical features (Supplementary Figure 7 (A)) and phase-derived coefficients (Supplementary Figure 7 (B)). Similar results to the first surrogate analysis conducted were obtained. When a random phase is introduced, the features are no longer able to statistically discriminate between the 4 classes, which reinforces the importance of the structural patterns contained in phase spectrum for particle differentiation tasks.

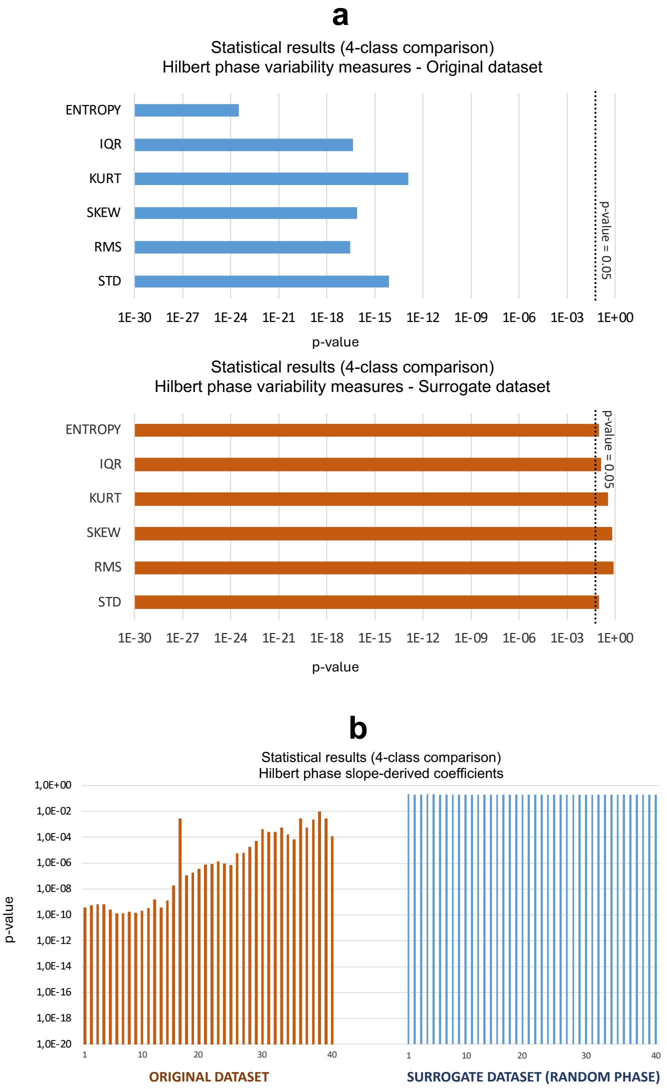

Supplementary Figure 7 Results obtained regarding 4-class statistical analysis of Hilbert phase slope derived features. a) Comparison between original and surrogate dataset of statistical features. b) Comparison between original and surrogate dataset of phase-derived coefficients. The statistical significance is lost, and the features are no longer able to discriminate between the 4 classes, which reinforces the crucial role of phase patterns contained in the back-scattering signals, for particle differentiation.

REFERENCES

[1] S. W. Smith, "The Scientist and Engineer's Guide to Digital Signal Processing". California Technical Pub, 1997.

[2] A. V. Oppenheim and R. W. Schafer, *Discrete-Time Signal Processing*. Prentice Hall, 1989.

[3] Z. N. Karam and A. V. Oppenheim, "Computation of the one-dimensional. unwrapped phase," *2007 15th Int. Conf. Digit. Signal Process. DSP 2007*, *IEEE*, pp. 304–307, 2007, doi: 10.1109/ICDSP.2007.4288579.

[4] J. M. Tribolet, "A New Phase Unwrapping Algorithm," *IEEE Trans. Acoust. Speech Signal Process.*, vol. 25, no. 2, pp. 170–177, 1977, doi: 10.1109/TASSP.1977.1162923.

[5] V. Cizek, “Discrete Hilbert transform,” *IEEE Trans. Audio Electroacoustics*, vol. 18, no. 4, pp. 340–343, Dec. 1970, doi: 10.1109/TAU.1970.1162139.

[6] Ronald Bracewell, *The Fourier Transform & Its Applications*, 3rd ed., vol. 31999. McGraw-Hill Science/Engineering/Math, 1999.

[7] G. Lancaster, D. Iatsenko, A. Pidde, V. Ticcinelli, and A. Stefanovska, “Surrogate data for hypothesis testing of physical systems,” *Phys. Rep.*, vol. 748, pp. 1–60, Jul. 2018, doi: 10.1016/j.physrep.2018.06.001.
