# Supplementary material for: Single-cell and extracellular nano-vesicles biosensing through phase spectral analysis of optical fiber tweezers back-scattering signals
Source: Commun Eng. 2024 Jul 10;3:97. doi: 10.1038/s44172-024-00240-1 (PMC11236981; doi:10.1038/s44172-024-00240-1)
Supplement: Supplementary file 4 — Lasing Reporting Summary [file 44172_2024_240_MOESM4_ESM.pdf]

Corresponding author(s): João Paulo Cunha

Lasing Reporting Summary

Nature Research wishes to improve the reproducibility of the work that we publish. This form is intended for publication with all accepted papers reporting claims of lasing and provides structure for consistency and transparency in reporting. Some list items might not apply to an individual manuscript, but all fields must be completed for clarity.

For further information on Nature Research policies, including our data availability policy, see Authors & Referees.

Experimental design

Please check: are the following details reported in the manuscript?

1. Threshold

Plots of device output power versus pump power over a wide range of values indicating a clear threshold

- [ ] Yes
- [x] No

A commercial laser (company: Lumics, reference: LU0980M500, wavelength: 980 nm, power: 500 mW, current: 750 mA) was used in the optical setup described in the manuscript, with an optical fiber tip output power of 10 ± 2 mW. Laser specifications such as threshold can be found in the manufacturers documentation, therefore such information was not included in the manuscript.

2. Linewidth narrowing

Plots of spectral power density for the emission at pump powers below, around, and above the lasing threshold, indicating a clear linewidth narrowing at threshold

- [ ] Yes
- [x] No

A commercial laser (company: Lumics, reference: LU0980M500, wavelength: 980 nm, power: 500 mW, current: 750 mA) was used in the optical setup described in the manuscript, with an optical fiber tip output power of 10 ± 2 mW. Laser specifications such as linewidth narrowing can be found in the manufacturers documentation, therefore such information was not included in the manuscript.

Resolution of the spectrometer used to make spectral measurements

- [ ] Yes
- [x] No

No spectral measurements were performed in the methodology described in the manuscript.

3. Coherent emission

Measurements of the coherence and/or polarization of the emission

- [ ] Yes
- [x] No

No measurements of coherence and/or polarization of the emission were performed in the methodology of the manuscript.

4. Beam spatial profile

Image and/or measurement of the spatial shape and profile of the emission, showing a well-defined beam above threshold

- [x] Yes
- [ ] No

In section B "Optical trapping and sensing" we provide references to previous works by our group were beam profile was analyzed. No measurements of emission profile were realized in the work described in the manuscript, as the optical setup was already stablished based on previous works, referenced in the manuscript.

5. Operating conditions

Description of the laser and pumping conditions
Continuous-wave, pulsed, temperature of operation

- [x] Yes
- [ ] No

Description of laser conditions is provided in section B "Optical trapping and sensing", where the reference of the commercial laser used in provided together with the wavelength, power, current and resulting optical fiber tip output power used to perform trapping of particles.

Threshold values provided as density values (e.g. W cm^{-2} or J cm^{-2}) taking into account the area of the device

- [ ] Yes
- [x] No

This information can be found in the manufacturer technical documentation.

6. Alternative explanations

Reasoning as to why alternative explanations have been ruled out as responsible for the emission characteristics
e.g. amplified spontaneous, directional scattering; modification of fluorescence spectrum by the cavity

- [ ] Yes
- [x] No

Not applicable in the optical setup described.

7. Theoretical analysis

Theoretical analysis that ensures that the experimental values measured are realistic and reasonable
e.g. laser threshold, linewidth, cavity gain-loss, efficiency

- [x] Yes
- [ ] No

In section B "Optical trapping and sensing" we provide references to previous works by our group were beam profile was evaluated via simulation procedures and theoretical analysis. No theoretical analysis was performed in the work described, as the optical setup was already stablished based on previous works, properly referenced in the manuscript.

8. Statistics

Number of devices fabricated and tested	☐ Yes ☒ No	Not applicable in the optical setup described.
Statistical analysis of the device performance and lifetime (time to failure)	☐ Yes ☒ No	This information can be found in the manufacturer technical documentation.
